# Oligonucleotide-capped nanoporous anodic alumina biosensor as diagnostic tool for rapid and accurate detection of *Candida auris* in clinical samples

**DOI:** 10.1080/22221751.2020.1870411

**Published:** 2021-03-11

**Authors:** Luis Pla, Sara Santiago-Felipe, María Ángeles Tormo-Mas, Alba Ruiz-Gaitán, Javier Pemán, Eulogio Valentín, Félix Sancenón, Elena Aznar, Ramón Martínez-Máñez

**Affiliations:** a Instituto Interuniversitario de Investigación de Reconocimiento Molecular y Desarrollo Tecnológico, Universitat Politècnica de València, Universitat de València Valencia, Spain; b CIBER de Bioingeniería, Biomateriales y Nanomedicina (CIBER-BBN), Madrid, Spain; c Unidad Mixta de Investigación en Nanomedicina y Sensores, Universitat Politècnica de València, Instituto de Investigación Sanitaria La Fe (IISLAFE), Valencia, Spain; d Grupo de Investigación Infección Grave, Instituto de Investigación Sanitaria La Fe (IISLAFE), Hospital Universitari i Politècnic La Fe, Valencia, Spain; e Servicio de Microbiología, Hospital Universitari i Politècnic La Fe, Valencia, Spain; f GMCA Research Unit, Departamento de Microbiología y Ecología, Universitat de Valencia, Valencia, Spain; g Unidad Mixta UPV-CIPF de Investigación en Mecanismos de Enfermedades y Nanomedicina, Universitat Politècnica de València, Centro de Investigación Príncipe Felipe, Valencia, Spain

**Keywords:** Nanoporous anodic alumina, oligonucleotide, molecular gates, *Candida auris*, biosensor, rapid diagnosis

## Abstract

*Candida auris* has arisen as an important multidrug-resistant fungus because of several nosocomial outbreaks and elevated rates of mortality. Accurate and rapid diagnosis of *C. auris* is highly desired; nevertheless, current methods often present severe limitations and produce misidentification. Herein a sensitive, selective, and time-competitive biosensor based on oligonucleotide-gated nanomaterials for effective detection of *C. auris* is presented. In the proposed design, a nanoporous anodic alumina scaffold is filled with the fluorescent indicator rhodamine B and the pores blocked with different oligonucleotides capable of specifically recognize *C. auris* genomic DNA. Gate opening modulation and cargo delivery is controlled by successful DNA recognition. *C. auris* is detected at a concentration as low as 6 CFU/mL allowing obtaining a diagnostic result in clinical samples in one hour with no prior DNA extraction or amplification steps.

## Introduction

*Candida auris* has emerged as a major pathogen due to the serious invasive infections it causes, and the various nosocomial outbreaks reported from different geographical locations since its first description in 2009 [1,2]. *C. auris* may cause some deep-seated infections and fungemia in risky population and approximately 50% of the isolated strains show resistance to, at least, two different classes of antifungal drugs. Besides, it may remain on surfaces for weeks and has high capacity of dissemination, which is unusual in other *Candida* species. The required time for *C. auris* acquisition is proposed to be less than 4 h in patients admitted to intensive care units (ICU), and skin colonization of patients can persist for months, favouring patient-to-patient transmission [3]. Also, it is worth mentioning that *C. auris* cannot be properly identified by commercial methods based on phenotypic techniques and it is frequently misidentified with phylogenetically related species, hindering right diagnosis and adequate treatment [4]. Given these difficulties, developing accurate, sensitive, and rapid detection methods for *C. auris* is vital to control the spread of the pathogen, treat infected patients quickly and appropriately, and prevent the appearance of new outbreaks.

As stated above, traditional methods for *C. auris* detection, based on morphological differentiation and biochemical tests, have resulted inappropriate for accurate recognition of this fungus in clinical laboratories as it is usually misidentified as *Rhodotorula glutinis*, *Candida haemoulonii*, *Saccharomyces cerevisiae*, or *Candida sake* [5]. Currently, more advanced identification techniques such matrix-assisted laser desorption ionization-time of flight mass spectrometry (MALDI-TOF MS) have emerged as alternative methods for correct *C. auris* identification. Else, molecular methods founded on sequencing and/or amplifying certain regions of the genome have been highly effective to discriminate *C. auris* from other intimately correlated species such as *Candida lusitaniae*, *Candida haemulonii*, and *Candida pseudohaemulonii* [4,6]. However, although these techniques usually show high sensitivity and specificity, they involve the use of expensive and specialized equipment, as well as experienced personnel, which limits their widespread application for clinical diagnosis.

During the last decade, biosensors technology has demonstrated an enormous potential for the detection of microorganisms in clinical applications [7,8]. Specifically, nanomaterials have resulted crucial for the development of some of such biosensors providing fast and accurate responses [9]. On that regard, the design of new hybrid bioorganic–inorganic materials allows to combine in a single entity the potential of nanomaterials with the recognition, selectivity and sensitivity properties of biomolecules[10]. Among nanomaterials utilized for the design of sensors, nanoporous anodic alumina (NAA) has become a suitable support due to its extraordinary features such as biocompatibility, the possibility to easily tune their surface and high loading capacity. Thanks to the aluminium oxide chemistry, it is possible to easily graft diverse (bio)molecules in the external surface of NAA acting as “molecular gates” [11]. When the support is loaded with an indicator and the modulation of its delivery is selectively triggered by a specific target, these materials become powerful sensing and diagnostic tools [12–15]. Nucleic acids (DNA, RNA or aptamers) are excellent examples of biomolecules able to act as molecular gates with extraordinary applications [16–18]. On that regard, several works combine the use of DNA with mesoporous supports to develop gated-nanodevices for applications in microorganism identification. For example, Bayramoglu et al. developed an oligonucleotide-functionalized gated nanosystem for the detection of *Salmonella* in milk samples [19]. Likewise, in a recent work, Ucak et al. achieved the recognition and inhibition of *Staphylococcus aureus* strains using teicoplanin encapsulated in an aptamer-gated PLGA nanoparticles [20]. In this field, we have developed an oligonucleotide-capped mesoporous support for *Mycoplasma fermentans* bacteria detection in cell culture media with a limit of detection of 20 DNA genome copies µL^–1^ [21]. More recently, we have successfully implemented gated systems for detecting *Candida albicans* in different human fluids, demonstrating the enormous potential that gated materials have in clinical settings [22].

In our proposed systems to detect *C. auris*, NAA supports are firstly loaded with the fluorescent reporter dye rhodamine B and then, capped with diverse oligonucleotide sequences that specifically hybridize with different regions of the *C. auris* genome. Oligonucleotides were selected from unique cell wall GPI-protein genes to obtain specific sequences that have no homology with sequences present in other species and they are just specific for *C. auris* improving the accuracy of the species identification [23]. The capping oligonucleotide blocks the pores and inhibits dye release. In the presence of *C. auris* genomic DNA, the capping oligonucleotide is displaced (due to favourable oligonucleotide-DNA hybridization), uncapping the pores and allowing dye delivery. A rapid response is observed from one of the gated NAA materials prepared, allowing an accurate *C. auris* detection in one hour with no prior DNA extraction or amplification steps in real clinical blood culture samples from infected patients, as well as in serum samples from healthy volunteers seeded with *C. auris* strains from different countries.

## Methods

### Oligonucleotides design

The design of the DNA probes was performed by in silico analysis of the translated reference genome of *C. auris* gene strain 6684, available in the NCBI database, to identify the *C. auris*-specific ORFs corresponding to the GPI cell wall proteins unique to *C. auris* (for additional information regarding the methodology employed on this subject, see reference [23]).

The specific sequences chosen to cap de pores were **O1**: 5′-TTT TGG GGG GTA CGC AAG GCG AAT CTA CCC GGG GGG TTT T-3′; **O2**: 5′-TTT TGG GGG GTC GCC ATT TTC TTT GTG GCG GGG GGG TTT T-3′; and **O3**: 5′-TTT TGG GGG GGC AGC ACT CGT GAG AGA ACT GGG GGG TTT T-3′. **O1** and **O2** contain a specific sequence which correspond to systematic ORF name QG37_01915 and **O3** to systematic ORF name QG37_05701. Oligonucleotides were acquired from Invitrogen by Thermo Fisher Scientific (Madrid, Spain).

### Synthesis of solids

To synthesize solid **S1**, 8 independent NAA supports of 2 mm of diameter were submerged in a rhodamine B solution in CH_3_CN (18.6 mg, 1 mm, 8 mL), which was mixed for 24 h to facilitate the loading of pores. Then surface functionalization was accomplished by the addition of (3-aminopropyl)triethoxysilane (1.32 mmol, 328 μL) and stirring the mixture during 6 h. Finally, the material was washed with a little amount of acetonitrile and dried overnight at room temperature.

To obtain the sensing solids **S2–S4**, **S1** was immersed in a solution of the corresponding aptamer sequence (**O1**–**O3**) in hybridization buffer (20 mM Tris-HCl, 37.5 mM MgCl_2_, pH 7.5). Optimization of capping conditions were carried out for each of the resulting gated materials to achieve the best performances. Amounts of capping oligonucleotide were 15 μL of **O1** (100 μM), 20 μL of **O2** (100 μM) and 15 μL of **O3** (100 μM). Final volume of reaction was stablished in 500 μL of hybridization buffer and mixtures were agitated at 37 °C for 60 min. Lastly, the obtained materials were rinsed with hybridization buffer to eliminate the unbounded oligonucleotide.

### Cargo quantification

To calculate the amount of the rhodamine B that can be loaded in the pores, a pair of independent supports of each solid **S2–S4** were submerged in 1 mL of hybridization buffer. Then, one of them was stirred at 90 °C during 60 min to force the opening of the pores and the maximum cargo release, and the other was maintained in agitation at 25 °C during 60 min as a control. The delivered fluorophore was measured at 575 nm (*λ*_exc_ = 555 nm), and the quantification of final released dye was undertaken using a calibration curve with different concentrations of rhodamine B. The experiment was done by triplicate.

### Detection protocol

The ability of the materials to detect *C. auris* was assessed by the fluorescence emission response of the reporter rhodamine B diffused from the inner mesoporous structure in the presence and in the absence of *C. auris* genomic DNA. For that, two independent supports of each material **S2–S4** were separately submerged in 900 µL of hybridization buffer each. Extracted DNA was pre-heated at 95 °C for 5 min and immediately cooled into an ice bath during 3 min to denature into single strains. Then, 100 μL of dehybridized DNA (1 ng μL^−1^) were added to one of the supports of each pair of solid **S2–S4** whereas 100 µL of hybridization buffer were transferred to the other. All solutions were stirred at 37 °C and aliquots were collected periodically. Released rhodamine B was detected by fluorescence spectroscopy at 575 nm (*λ*_exc_ = 555 nm).

### Quantification curve of genomic DNA

The response of the solids **S2–S4** to different concentrations of *C. auris* genomic DNA was studied. For that, seven independent supports of each material **S2–S4** were submerged in a solution containing 100 µL of 10-fold dilutions of dehybridized DNA (1–10^−6^ ng μL^−1^) and volume was completed until 1 mL with hybridization buffer. Solutions were stirred at 37 °C and released rhodamine B was determined at 575 nm (*λ*_exc_ = 555 nm) after 60 min.

### Amplification Assay

In order to calculate the amplification of the signal, two individual **S4** solids were submerged in 900 μL of hybridization buffer. Then, 100 µL of dehybridized DNA (1 ng μL^−1^) was added to one of them and 100 µL of hybridization buffer was added to the other. Both solutions were stirred at 37 °C during 60 min and the delivered fluorophore was observed at 575 nm (*λ*_exc_ = 555 nm). Quantification of finally released dye was undertaken using a calibration curve with different concentrations of rhodamine B, to then directly correlate with the concentration of genomic DNA from *C. auris* that opened the system.

### Selectivity

To evaluate de selectivity of the system, dye release experiments were carried out by adding to seven independent supports of each material **S2–S4** 100 μL of dehybridized DNA (1 ng μL^−1^) from other *Candida* species (*C. albicans, C. glabrata, C. parapsilosis, C. tropicalis, C. pseudohaemulonii, C. haemulonii, C. intermedia*, and *C. lusitaniae*). In the same experiment, 100 µL of DNA from *C. auris* at 1 ng μL^−1^ was used as a positive control and 100 µL of hybridization buffer as a negative control. The final volume was completed to 1 mL with hybridization buffer and mixed for 60 min at 37 °C. Delivered rhodamine B was determined by fluorescence (*λ*_exc_ = 555 nm, *λ*_em_ = 585 nm).

### Detection of *C. auris* in real competitive media

The potential use of the sensing material **S4** to detect different concentrations of *C. auris* in more realistic samples was tested. Thus, *C. auris* cells were cultured in YPD agar medium (Difco®) at 37 °C to prepare a final 0.5 McFarland solution (10^6^ CFU mL^−1^). Then, human blood from healthy donors was collected in a “gel-and-clot activator tube” for serum separation and then was artificially inoculated with different concentrations of *C. auris* (10^4^–0 CFU mL^−1^). In a next step, serum was obtained by centrifugation of blood clots at 3000*g* for 10 min. Finally, 500 µL of each serum were added to five independent **S4** supports submerged in 500 µL of hybridization buffer. After 60 min at 37 °C, the delivered rhodamine B was determined according to the obtained fluorescence emission at 575 nm (*λ*_exc_ = 555 nm).

In other experiment, the ability of the **S4** nanosensor to identify *C. auris* strains from different countries was evaluated. For that, fourteen serums from healthy donors were artificially inoculated with a concentration of 10^2^ CFU mL^−1^ of *C. auris* strains from eight different geographical locations (Korea, Japan, India, Venezuela, Kuwait, Oman, Colombia and Spain). In a next step, 14 independent **S4** supports were submerged in 500 µL of each serum and 500 µL of hybridization buffer was added to each one. After 60 min at 37 °C, the delivered rhodamine B was determined according the obtained fluorescence emission at 575 nm (*λ*_exc_ = 555 nm).

### Validation in real clinical samples

The performance of the solid **S4** was evaluated for its application in the recognition of *C. auris* in real clinical samples. For that, 22 blood culture samples from infected (17 samples) and non-infected (5 samples) patients from Hospital Universitari i Politècnic La Fe were tested by adding 500 µL of the blood culture and 500 µL of hybridization buffer to each independent **S4** support. Finally, the diffused rhodamine B was determined after 60 min at 37 °C by measuring fluorescence emission at 575 nm (*λ*_exc_ = 555 nm)

## Results and discussion

### Synthesis and characterization of gated NAA

**Figure 1.**
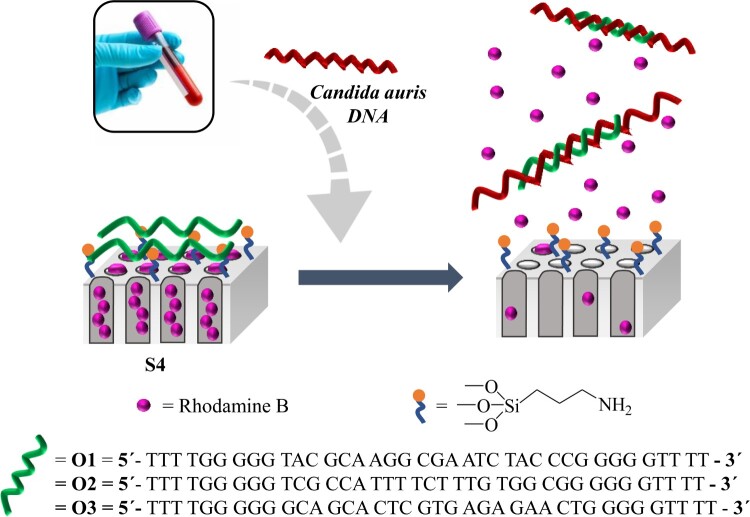
Schematic representation of the gated NAA support and the selective uncapping and dye delivery in the presence of *C. auris* genomic DNA.

In our proposed system, pores of NAA were filled with the fluorescent reporter rhodamine B and the outer surface was chemically modified by the attachment of (3-aminopropyl)triethoxysilane, to give support **S1**. Finally, oligonucleotides **O1**–**O3** were used to cap the pores through strong electrostatic and hydrogen bonding interactions between protonated aminopropyl moieties on the NAA surface and the negatively charged oligonucleotides. This resulted in the preparation of the capped supports **S2**–**S4**, respectively. As stated above, *C. auris* genomic DNA is expected to selectively induce the aperture of the pores, allowing the release of the encapsulated fluorophore (Figure 1).

The raw NAA material and **S1**, **S2, S3** and **S4** were characterized by FESEM and EDX analyses (see supplementary material for details). Commercially available NAA supports are composed of anodic aluminium oxide films grown on a 0.1 mm thick aluminium layer with a pore density of 9 × 10^11^ cm^−2^. Pores entrance has a funnel-like shape which progressively shifts from a larger size (20–30 nm) at the top of the funnel to a 5 nm size at the end. This transition reaches a profundity of ca. 15 nm, and below this depth, the pore configuration becomes uniform along 10 µm. FESEM images of the starting NAA scaffold confirmed the porous structure. Moreover, images of **S2–S4** evidence the presence of a dense organic layer which cap the pores and attributed to the presence of the capping oligonucleotides (Figure S-1).

The successive steps in the preparation of the final gated materials (i.e. **S2**, **S3** and **S4**) were followed by energy dispersive X-ray spectroscopy (Table S-1). As a result of dye loading on the pores and anchoring of 3-aminopropyl groups on the NAA surface, solid **S1** shows a high carbon content (C/Al 1.06) and the presence of nitrogen (N/Al 0.33). During the preparation of **S2**–**S4**, some dye is lost before the oligonucleotides are able to cap the pores. As a result, the C/Al ratio decreases and there is a slight increase in nitrogen content attributed to the oligonucleotide. The presence of the oligonucleotides is also demonstrated in **S2**–**S4** by the detection of phosphorus that is neither observed in the starting NAA nor in the **S1** support. In addition, from extraction experiments, the concentration of rhodamine B in the final materials was calculated to be ca. 4 mg/g NAA.

### Release assays

**Figure 2.**
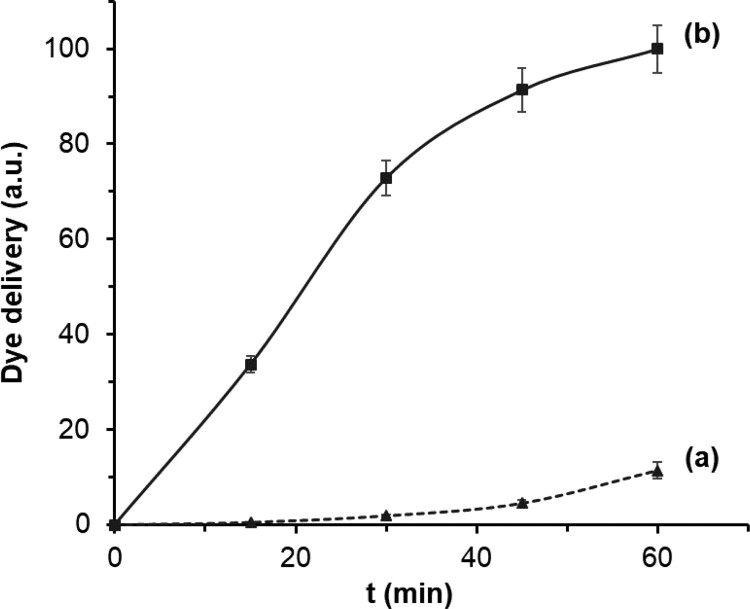
Rhodamine B delivery profile from **S4** in the absence (a) and in the presence (b) of 100 ng of denatured genomic DNA of *C. auris* in hybridization buffer 20 mM Tris–HCl, 37.5 mM MgCl_2_, pH 7.

Rhodamine B delivery kinetics from solids **S2**–**S4** was studied to confirm the specific opening of the gated materials in the presence of *C. auris* genomic DNA. To carry out this study, two independent gated supports of each solid (i.e. **S2**–**S4**) were separately submerged in hybridization buffer. Then, 100 μL of previously denatured genomic DNA (1 ng μL^−1^) were added to one of the solutions and 100 µL of buffer was added to the other. To quantify the amount of the delivered dye from the pores to the aqueous phase, the fluorescence of the supernatant solution was measured at predetermined times (see supplementary material for details). As an example, Figure 2 shows the rhodamine B delivery profile from solid **S4** in the absence and presence of *C. auris* genomic DNA. **S4** in hybridization buffer delivers a very low amount of dye (less than 10% of the maximum dye delivered), which indicates a tight pore closure (Figure 2, curve a). On the contrary, when *C. auris* genomic DNA is present a much larger amount of rhodamine B is released (10-fold at 60 min, Figure 2, curve b). The performance of the nanomaterial demonstrates that *C. auris* genomic DNA is able to uncap the pores and induce cargo delivery as represented in Figure 1.

Solids **S2** and **S3** offered a similar response, demonstrating tight pore closure and dye delivery when *C. auris* DNA is present (Figure S-2). However, material **S4** showed a better performance, providing a quicker response and higher dye release after 60 min. These differences are most likely indicative of a more efficient recognition and hybridization between **O3** and *C. auris* DNA when compared with **O1** and **O2**.

### Sensitivity and specificity studies

In these experiments, solids **S2**–**S4** were treated with 100 μL of different concentrations of *C. auris* genomic DNA (1–10^−6^ ng μL^−1^) in hybridization buffer following a similar protocol as shown above. After *C. auris* genomic DNA addition for each point, the emission in the solution was measured after 60 min. For all materials, the quantity of released dye increases as a function of the DNA concentration, which agrees with the above proposed uncapping protocol (Figure S-3). Limits of detection of 0.5, 0.5, and 0.3 pg/µL were calculated for **S2**, **S3**, and **S4**, respectively (Table S-2). Remarkably, these LODs are in the range of those usually reported by instrumental techniques used for *C. auris* detection (MALDI-TOF MS, RT–PCR or T2 Magnetic resonance); nevertheless, our method is faster, simpler, and do not require specialized instrumentation or database [24–29]. The low limits of detection we observed are most likely due to a favourable interaction between the capping oligonucleotides and the *C. auris* genomic DNA and also due to the intrinsic signal amplification when used gated materials. Thus, it has been reported that in these gated systems a unique analyte molecule is able to induce the delivery of a large amount of dye molecules when the pores are opened. In fact, recent works demonstrated that the number of delivered dye molecules can be 10^4^–10^11^ per molecule of DNA [23,30]. In this work, it has been estimated that 1 ng of genomic DNA was capable of release an average of 1.5 × 10^11^ molecules of rhodamine B, demonstrating a high signal amplification capacity.

**Figure 3.**
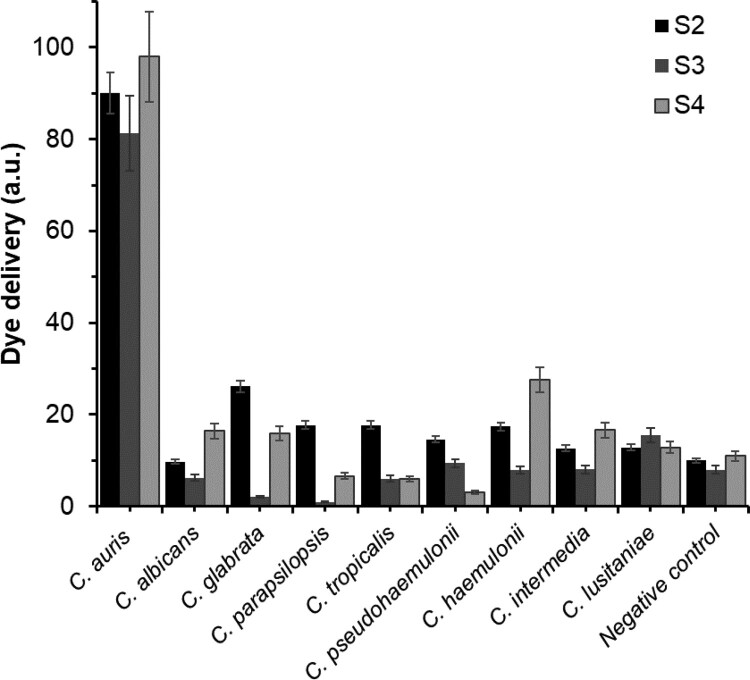
Rhodamine B delivery from solids **S2** (black), **S3** (dark grey) and **S4** (light grey) in the presence of 1 ng μL^−1^ of genomic DNA of *C. auris*, *C. albicans*, *C. glabrata*, *C. parapsilopsis*, *C. tropicalis*, *C. pseudohaemulonii*, *C. haemulonii*, *C. intermedia*, and *C. lusitaniae* and 100 µL of hybridization buffer as a negative control. Fluorescence was measured after 60 min in hybridization buffer at pH 7.5.

In a step forward, the selectivity of **S2**, **S3**, and **S4** to *C. auris* was tested (Figure 3). In these experiments, the response of each solid was assessed in the presence of 100 μL of dehybridized DNA (1 ng μL^−1^) from *C. auris* and eight other *Candida* species (Table S-3) in 900 μL of hybridization buffer. As it can be observed, only *C. auris* genomic DNA was able to trigger a notable rhodamine B release while other *Candida* species induced poor uncapping and cargo delivery, indicating a high selective response of the nanomaterials **S2**–**S4** for *C. auris*.

### Detection of *C. auris* in competitive media and inoculated samples

**S4** was also tested in a more competitive medium and cargo release experiments with **S4** were carried out in serum samples containing different concentrations of *C. auris*. For that, human blood, collected in a “gel-and-clot activator tube” for serum separation, was artificially inoculated with serial dilutions of *C. auris* from 10^4^ to 0 CFU mL^−1^. Then, serum was extracted by centrifugation of the blood clots at 3000*g* for 10 min and the response of **S4** was tested in the presence of 500 μL of serum mixed with 500 μL of hybridization buffer. Dye delivered was measured after 60 min. It was found that dye release was proportional to the *C. auris* concentration in blood samples and a LOD of 6 CFU mL^−1^ was calculated based on the intersection point of the two slopes of the represented curve (Figure S-4). This value is within the low range of expected concentrations in infected human fluids (1–10^3^ CFU mL^−1^) and it is similar to those obtained by common commercially available methodologies for *C. auris* diagnosis, demonstrating the capability of the method for its use in clinical applications without the need of previous culture, sample treatment or amplification steps. Note that we did not carry out steps such as cell lysis or DNA extraction, yet we are able to detect the presence of *C. auris*. This agrees with previous works that demonstrated that genomic DNA is released to the extracellular medium and can be detected [22].

In a further study, we validated if independent and genetically diverse clades were successfully recognized by **S4**. Fourteen serum samples artificially inoculated with *C. auris* strains from eight different countries were analysed. For that, an individual support **S4** was immersed in a mixture containing 500 μL of hybridization buffer and 500 μL of the inoculated serum sample. After 60 min at 37 °C, delivered rhodamine B was determined by fluorescence spectroscopy. The sample was considered positive when the fluorescence intensity at 585 nm (*λ*_exc_ = 555 nm) was higher than the average fluorescence of negative controls plus three times their standard deviation. All tested strains were previously identified positively by sequencing of internal transcribed spacer (ITS). Results showed that **S4** is able to detect isolates from all countries except one from Colombia (Table 1). However, it is worth mentioning that further PCR studies confirmed that this Colombian isolate presented a rare genetic difference in the coding region of the QG37_05701 gene and do not contain the specific target sequence used in this experiment [23], what can explain the obtained false negative. In previous work from Ruiz-Gaitán et al., this specific isolate was also giving negative results when analysed by PCR using QG37_05701 primers. This fact was due to the lack of the whole QG37_05701 in two patients from a specific hospital whereas other Colombian isolates do not. The results suggested a genomic plasticity and the capacity of *C. auris* for horizontal transmission. This particular QG37_05701 genotype seems rare as it observed just in that two Colombian isolates and not in others from the same country. This special false negative confirms the high accuracy of the gated material. Also note that this false negative would have not been obtained using solids **S2** or **S3**, which are designed from QG37_01915 gene and further work is being carried out to optimize our sensing protocol using other oligonucleotides as caps.

**Table 1.** Results from serum samples inoculated with *C. auris* from different geographical locations analysed using the gated nanomaterial **S4** and the reference procedure.

# Isolate	Country	Reference method^a^	**S4**^b^
1	Korea	+	+
2	Japan	+	+
3	India	+	+
4	Venezuela	+	+
5	Kuwait	+	+
6	Oman	+	+
7	Colombia	+	**−**
8	Spain	+	+
9	Spain	+	+
10	Spain	+	+
11	Spain	+	+
12	Spain	+	+
13	Spain	+	+
14	Spain	+	+

^a^ Strains were re-confirmed by molecular sequencing.

^b^ Positive (+) was considered when the fluorescence intensity at 585 nm (*λ*_exc_ = 555 nm) was higher than the average fluorescence of the negative controls plus three times their standard deviation.

Clonality of *C. auris* isolates from South Africa, Brazil and South Korea have previously been identified using amplifying techniques like fragment length polymorphism (AFLP) and multilocus sequence typing (MLST). Despite their high discriminatory power and good reproducibility, these complex methods are high cost and their use is usually restricted to research laboratories [31–33]. On the other hand, molecular techniques like whole-genome sequencing (WGS) has demonstrated to provide enough information to discern between *C. auris* isolated strains and their phylogenetic relationships using single nucleotide polymorphism (SNP) analysis [34]. In this context, the portable MinION sequencer (Oxford Nanopore Technologies, UK) has been successfully employed for analysing the molecular epidemiology of Ebola, Zika, and *C. auris* outbreaks [35]. Alternatively, solid **S4** has proved its ability to discriminate *C. auris* isolates from different countries in 60 min, with a simple and low-cost procedure, avoiding DNA extraction or amplification steps, what makes it suitable for use in point-of-care detection systems.

### Validation in real clinical samples

Due to the increase of invasive infections by *C. auris* around the world, the development of fast, sensitive, and reliable diagnosis techniques becomes an important challenge [36,37]. In this scenario, **S4** was assessed as diagnostic tool as an alternative to existing techniques for *C. auris* detection in clinical samples from infected patients. The most usual method followed by hospitals to detect candidemia is blood culture automatized systems and subsequent identification of the isolates by biochemical, phenotypic, and proteomic techniques. Using this procedure, the average time between sample collection and final identification is 3–5 days.

In our study, 22 blood culture samples of infected and non-infected patients from Hospital Universitari i Politècnic La Fe were first analysed by the reference method. Clinical and demographic data from the patients included in the study can be seen in Table S-4 **(**supplementary material**)**. By this way, 17 samples were confirmed as positive and five resulted negative. In parallel, the same samples were analysed by our proposed method following a blinded procedure (see supplementary material for more details). For that, 22 individual **S4** supports were immersed with 500 μL of the sample and 500 μL of hybridization buffer, and fluorescence was measured after 60 min. As above, a sample was considered positive when the fluorescence intensity at 585 nm was higher than the average fluorescence of the negative controls plus three times their standard deviation. A good correlation between the standard method for the diagnosis of *C. auris* and our procedure was observed; a sensitivity of 85%, a specificity of 100%, and positive and negative predictive values of 100% and 62.5%, respectively, were calculated (Table 2). It should be considered that false negatives may occur because samples came from blood cultures stored at –20 °C for more than 6 weeks, that may induce DNA fragmentation in less–high-molecular-weight DNA sequences, interfering in the DNA–DNA hybridization[38].

**Table 2.** Results from blood culture samples analysed using the gated nanomaterial **S4** and the reference procedure.

# Sample	Reference method^a^	**S4**^b^
1	−	−
2	−	−
3	−	−
4	−	−
5	−	−
6	+	+
7	+	+
8	+	−
9	+	+
10	+	+
11	+	+
12	+	−
13	+	+
14	+	+
15	+	+
16	+	+
17	+	+
18	+	+
19	+	+
20	+	+
21	+	−
22	+	+

^a^ Positive (+) was considered when any *C. auris* colony is isolated from blood culture samples.

^b^ Positive (+) was considered when the fluorescence intensity at 585 nm (*λ*_exc_ = 555 nm) was higher than the average fluorescence of the negative controls plus three times their standard deviation.

The identification of *C. auris* in biological samples is an important challenge in the diagnostic field. Current methods are based on molecular techniques such as DNA amplification or sequencing, and more recently, based on MALDI-TOF MS. Despite these techniques provide high sensitivity and specificity, they usually require expensive and specialized equipment and personnel, which is outside the reach of most clinical laboratories. On the other hand, biosensors based on oligonucleotide-capped NAA have been shown numerous advantages: (i) they have great versatility, both in the cargo and in the capping DNA sequence; (ii) the cost of preparation and testing is considerably low comparing to other standardized techniques; (iii) the required equipment is simple, common, and affordable for majority of laboratories, and (iv) the analysis procedure is faster, simpler and avoids sample treatments such as DNA extraction or amplification. Therefore, it is noteworthy the high competitiveness of our gated probe, being a promising alternative for the fast and precise detection of *C. auris*.

## Conclusions

Fast and reliable diagnosis of *C. auris* infections is critical to carry out appropriate treatment and to control fungal outbreaks and new approaches that allow to identify *C. auris* in a simple and rapid manner is crucial in the clinical field. In this study, an innovative methodology for *C. auris* detection by using a fluorogenic nanosensor is presented. The system consists of NAA mesoporous supports loaded with a fluorophore and capped with an oligonucleotide. In the presence of *C. auris* genomic DNA the capping oligonucleotide is displaced from the NAA surface to hybridize *C. auris* genomic DNA resulting in pore opening and delivery of the entrapped fluorophore. All capped NAA show excellent selectivity and sensitivity. Particularly, solid **S4** capped with oligonucleotide **O3** resulted to be the most effective systems reaching higher and faster dye release, and a lower limit of detection (0.3 pg µL^−1^). The gated material **S4** is tested with human blood inoculated with different amounts of *C. auris* cells and it successfully recognize *C. auris* isolates from eight different countries in artificially inoculated serum samples. Moreover, the method allows an accurate detection of *C. auris* in blood culture samples from infected patients in 60 min without the need of previous sample treatment or amplification steps. The proposed technique has great potential as diagnostic tool for *C. auris* detection in clinical samples.

## Supplementary Material

Revised_Supplementary_Material_editable.docx
