# Supplementary material for: Oligonucleotide-capped nanoporous anodic alumina biosensor as diagnostic tool for rapid and accurate detection of Candida auris in clinical samples
Source: Emerg Microbes Infect. 2021 Mar 11;10(1):407–15. doi: 10.1080/22221751.2020.1870411 (PMC7954474; doi:10.1080/22221751.2020.1870411)
Supplement: Revised_Supplementary_Material_editable.docx [file TEMI_A_1870411_SM0220.docx]

# SUPPLEMENTARY MATERIAL

**Oligonucleotide-capped nanoporous anodic alumina biosensor as diagnostic tool for rapid and accurate detection of *Candida auris* in clinical samples.**

Luis Pla,^1,2,3,^ M. Ángeles Tormo-Mas,^4^ Alba Ruiz-Gaitan,^4^ Javier Pemán,^4,7^ Eulogio Valentín,^4,5^ Félix Sancenón,^1,2,3,6^ Elena Aznar,^1,2,3,6,*^ Ramón Martínez-Máñez,^1,2,3,6*^ Sara Santiago-Felipe^1,2,3^

^1^ CIBER de Bioingeniería, Biomateriales y Nanomedicina (CIBER-BBN)

^2^ Instituto Interuniversitario de Investigación de Reconocimiento Molecular y Desarrollo Tecnológico, Universitat Politècnica de València, Universitat de València, Camino de Vera s/n, 46022, Valencia, Spain

^3^ Unidad Mixta de Investigación en Nanomedicina y Sensores. Universitat Politècnica de València, Instituto de Investigación Sanitaria La Fe, Valencia, Spain

^4^ Grupo de Investigación Infección Grave. Instituto de Investigación Sanitaria La Fe, Hospital Universitari i Politècnic La Fe, Valencia, Spain

^5^ GMCA Research Unit, Departamento de Microbiología y Ecología, Universitat de Valencia, Spain

^6^ Unidad Mixta UPV-CIPF de Investigación en Mecanismos de Enfermedades y Nanomedicina. Universitat Politècnica de València, Centro de Investigación Príncipe Felipe, Valencia, Spain

^7^ Servicio de Microbiología, Hospital Universitari i Politècnic La Fe, Valencia, Spain

# Methods

## *General techniques*

Energy Dispersive X-ray spectroscopy (EDX) analyses and field emission scanning electron microscopy (FESEM) were done in a ZEISS Ultra 55 microscope.

Fluorescence spectroscopy measurements were executed on a Synergy H1 microplate reader (BioTek, Winooski, VT, USA). Culture centrifugation was undertaken in an Accuspin Micro 17 centrifuge (Fisher Scientific).

## *Chemicals, scaffolds and oligonucleotides*

(3-aminopropyl)triethoxysilane, hydrochloric acid, rhodamine B, triethylamine (TEA) and tris(hydroxymethyl)aminomethane (TRIS) were acquired from Sigma-Aldrich Química (Madrid, Spain). Nanoporous anodic alumina supports were commercially obtained from InRedox® (CO, USA).

## *Strains and growth conditions*

Distinct collections of *Candida* strains were tested: i) *C. auris* isolates obtained from clinical samples of patients at Hospital Universitari i Politècnic La Fe; ii) representative isolates of several global published outbreaks (Korea, Japan, India, Venezuela, Kuwait, Oman and Colombia); iii) a collection of other *Candida* species, including the most prevalent pathogens (*C. albicans, Candida glabrata, Candida parapsilosis* and *Candida. tropicalis*) and also the *C. auris* closest related species (*C. lusitaniae, C. haemulonii, C. pseudohaemulonii and C. intermedia*). Many strains employed were provided from the American Type Culture Collection (ATCC) (Table S-1, Supporting Information). All isolates were growth at 37ºC on Yeast Extract-Peptone-Dextrose (YPD) Agar (BD® Difco®). The yeast concentration was determined by preparing a 0.5 McFarland solution in hybridization buffer, corresponding to 10^6^ CFU mL^-1^.

## *DNA extraction*

The DNA extraction was performed using the procedure developed by Hoffman *et al*.[3] Briefly, yeast cells were grown on YPD agar for 24 h at 37 ºC. Then, isolated colonies were inoculated to 10 mL of YPD broth (1 g L^-1^ of yeast extract, 2 g L^-1^ of casein peptone, 2 g L^-1^ of dextrose) and incubated with shaking for 24 h at 37 ºC. In a next step, 1.5 to 3 ml of the culture were centrifuged at 0.9 g for 5 min and 0.3 g of RNase-free glass beads and 200 µl of lysis solution (Triton 2%, SDS 1%, NaCl 10 mM, EDTA 1 mM, Tris 10 mM, pH 8.0) were added to the pellet. Finally, the solution was vortexed for 3 min and 300 µl of Trizol and 100 µl of chloroform were added to the mixture. The extracted DNA was quantified and stored at -70 ºC.

## *Analysis of clinical samples by the reference method*

Samples were analysed by the Principles of Good Laboratory Practice (GLP). In a first step, samples for blood culture were obtained under aseptic conditions and processed by a conventional automated system (BacT/ALERT®VIRTUOTM, bioMérieux, Marcy

l’Etoile, France) until they are detected as positive (10^6^-10^8^ CFU mL^-1^). Cultures positivization times varies between yeast species and range from 2.2 to 184 hours. Specifically, *C. auris* presents a time of growth of around 30 hours. After that, phenotypic colony features were examined after 48 hours of incubation in Sabouraud dextrose agar (Becton Dickinson,Baltimore, USA) and CHROMagar Candida (Chromagar, Paris, France). After that, all *Candida* isolates were identified by biochemical characteristics (AuxaColor2TM, BioRad Laboratories, Marnes la Coquette, France) and proteomic profiling (VITEK MS IVD, bioMérieux, Marcy l’Etoile, France) in agreement to manufacturer’s instructions, what may involve additional 24-48 hours. Finally, the identification of *C. auris* was confirmed by molecular sequencing of

internal transcribed spacer (ITS) using the primers ITS4-ITS5 as previously described.[4]

## *Field emission scanning electron microscopy (FESEM) images*


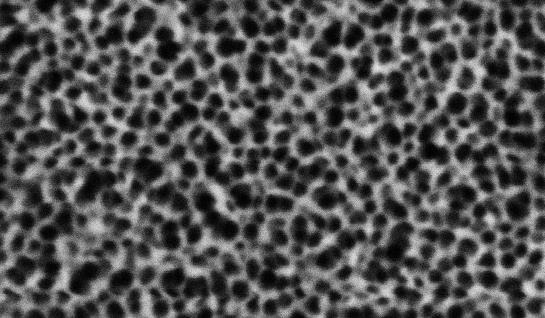


**a)**

**100 nm**


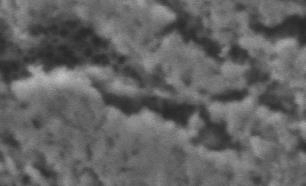


**b)**

**100 nm**

Figure S-1. Characteristic images from FESEM microscope of (a) NAA support, and (b)

**S4**.

## *Energy Dispersive X-ray spectroscopy (EDX) analyses*

Table S-1. Atomic elemental ratios in the different prepared solids.

|  | **C/Al** | **N/Al** | **P/Al** |
| --- | --- | --- | --- |
| **S1** | 1.06 | 0.33 | - |
| **S2** | 0.66 | 0.38 | 0.04 |
| **S3** | 0.55 | 0.36 | 0.03 |
| **S4** | 0.62 | 0.38 | 0.03 |

## *Release profiles*


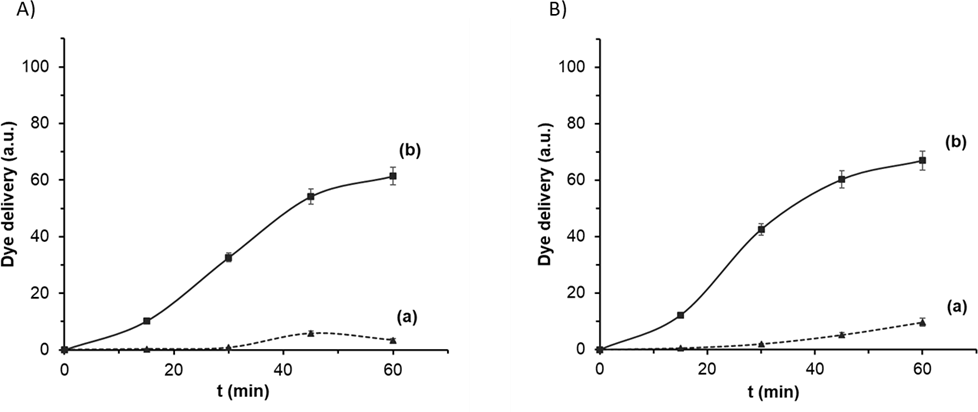


Figure S-2. Release profile of rhodamine B from solids **S2** (A) and **S3** (B) in the absence

(a) and in the presence (b) of 100 ng/mL of dehybridized genomic DNA of *C. auris* in hybridization buffer (20 mM Tris-HCl, 37.5 mM MgCl2, pH 7.5).

## *Limits of Detection*


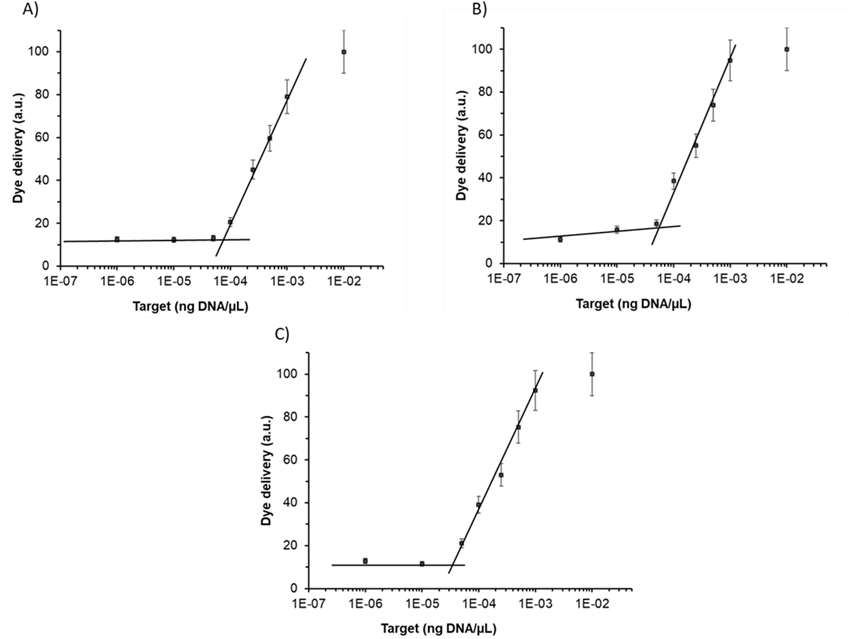


Figure S-3. Release of rhodamine B from solids (A) **S2**, (B) **S3** and (C) **S4**, in the presence of different concentrations of dehybridized genomic DNA from *C. auris* in hybridization buffer (20 mM Tris-HCl, 37.5 mM MgCl2, pH 7.5).

Table S-2. Limits of detection of solids **S2**-**S4**.

| **Oligonucleotide** | | **Limit of detection**  **(pg/µL)** |
| --- | --- | --- |
| **S2** | **O1** | 0.5 |
| **S3** | **O2** | 0.5 |
| **S4** | **O3** | 0.3 |


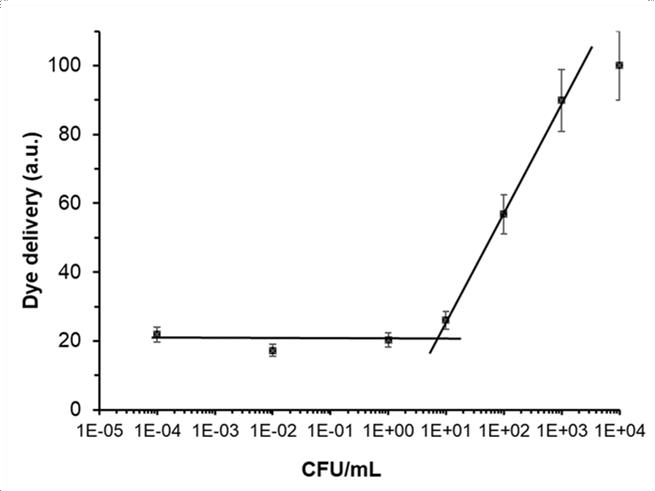


Figure S-4. Rhodamine B delivery from solid **S4** in blood samples inoculated with different concentrations of *C. auris*.

## *Yeast strains*

Table S-3. Yeast strains

| **Yeast** | **Strain (ATTC)** |
| --- | --- |
| *Candida auris* | VPCI 473/P/13 |
| *Candida albicans* | SC-5314 |
| *Candida glabrata* | ATCC 2001 |
| *Candida parapsilopsis* | ATCC 563 |
| *Candida tropicalis* | BIOCAN-10 |
| *Candida pseudohaemulonii* | CBS12370 |
| *Candida haemulonii* | CBS 12371 |
| *Candida intermedia* | ATCC 20178 |
| *Candida lusitaniae* | CR-135 |

## *Validation in real clinical samples*

Table S-4. Information about the patients

| **Patient** | **Age, Gender** | **Ward/Critical unit** | **Diagnosis** | **Treatment** |
| --- | --- | --- | --- | --- |
| 1 | 68y, Male | Hepatology | Hepatic cholangiocarcinoma | Anidulafungin |
| 2 | 68y, Female | Digestive surgery | Bladder cancer | Anidulafungin |
| 3 | 75y, Male | Thoracic surgery | Traumatism | Anidulafungin |
| 4 | 68y, Male | Digestive surgery | Urothelial carcinoma | Micafungin |
| 5 | 79y, Male | Critical care surgery | Surgical wound infection | Anidulafungin |
| 6 | 63y, Female | Internal medicine | Duodenal ulcer | Anidulafungin |
| 7 | 41y, Male | Critical care surgery | Polytrauma | Anidulafungin |
| 8 | 70y, Female | Internal medicine | Surgical wound infection | Anidulafungin |
| 9 | 17y, Male | Critical care surgery | Pneumothorax | Anidulafungin |
| 10 | 49y, Male | Internal medicine | Pneumonia | Anidulafungin |
| 11 | 60y, Male | Critical care surgery | Dilated cardiomyopathy | Anidulafungin |
| 12 | 20y, Male | Critical care surgery | Polytrauma | Anidulafungin |
| 13 | 44y, Male | Internal medicine | Cardiogenic shock | Anidulafungin + Amphotericin B |
| 14 | 45y, Male | Medical critical care unit | Cardiogenic shock | Anidulafungin + Amphotericin B |
| 15 | 65y, Male | Critical care surgery | Solid organ transplant | Caspofungin |
| 16 | 71y, Male | Critical care surgery | Aneurysm | Anidulafungin |
| 17 | 50y, Male | Critical care surgery | Sepsis | Anidulafungin |
| 18 | 80y, Male | Critical care surgery | Mitral-aortic valve disease | Anidulafungin |
| 19 | 21y, Male | Critical care surgery | Polytrauma | Anidulafungin |

# References

1. Taylor S, Wakem M, Dijkman G, et al. A practical approach to RT-qPCR- Publishing data that conform to the MIQE guidelines. Methods. 2010;50:S1-S5.
2. Bustin SA, Benes V, Garson JA, et al. The MIQE guidelines: Minimum information for publication of quantitative real-time PCR experiments. Clin Chem. 2009;55:611–22.
3. Hoffman CS, Winston F. A ten-minute DNA preparation from yeast efficiently releases autonomous plasmids for transformaion of Escherichia coli. Gene 1987;57:267–72.
4. Bellemain E, Carlsen T, Brochmann C, et al. ITS as an environmental DNA barcode for fungi: An in silico approach reveals potential PCR biases. BMC Microbiol. 2010;10:189.
